# Stakeholders’ views on the ethical challenges of pragmatic trials investigating pharmaceutical drugs

**DOI:** 10.1186/s13063-016-1546-3

**Published:** 2016-08-22

**Authors:** Shona Kalkman, Ghislaine J. M. W. van Thiel, Diederick E. Grobbee, Anna-Katharina Meinecke, Mira G. P. Zuidgeest, Johannes J. M. van Delden, on behalf of Work Package 3 of the IMI GetReal Consortium

**Affiliations:** 1 Department of Medical Humanities, Julius Center for Health Sciences and Primary Care, University Medical Center Utrecht, PO Box 85500, 3508 GA Utrecht, The Netherlands; 2 Department of Epidemiology, Julius Center for Health Sciences and Primary Care, University Medical Center Utrecht, Utrecht, The Netherlands; 3 GMACS GHEOR, Bayer Pharma AG, Wuppertal, Germany

**Keywords:** Pragmatic clinical trial, Comparative effectiveness, Research ethics, Qualitative study, Drug research

## Abstract

**Background:**

We explored the views of key stakeholders to identify the ethical challenges of pragmatic trials investigating pharmaceutical drugs. A secondary aim was to capture stakeholders’ attitudes towards the implementation of pragmatic trials in the drug development process.

**Methods:**

We conducted semistructured, in-depth interviews among individuals from different key stakeholder groups (academia and independent research institutions, the pharmaceutical industry, regulators, Health Technology Assessment (HTA) agencies and patients’ organizations) through telephone or face-to-face sessions. Interviews were structured around the question “what challenges were experienced or perceived during the design, conduct and/or review of pragmatic trials.” Respondents were additionally asked about their views on implementation of pragmatic trials in the drug development process. Thematic analysis was used to identify the ethically relevant features across data sets.

**Results:**

We interviewed 34 stakeholders in 25 individual sessions and four group sessions. The four perceived challenges of ethical relevance were: (1) less controlled conditions creating safety concerns, (2) comparison with usual care potentially compromising clinical equipoise, (3) tailored or waivers of informed consent affecting patient autonomy, and (4) minimal interference with “real-world” practice reducing the knowledge value of trial results.

**Conclusions:**

We identified stakeholder concerns regarding risk assessment, use of suboptimal usual care as a comparator, tailoring of informed consent procedures and ensuring the social value of pragmatic trials. These concerns increased when respondents were asked about pragmatic trials conducted before market authorization.

**Electronic supplementary material:**

The online version of this article (doi:10.1186/s13063-016-1546-3) contains supplementary material, which is available to authorized users.

## Background

The majority of randomized controlled trials (RCTs) in drug research are explanatory trials that focus on drug safety and efficacy [1, 2]. They are currently accepted as the highest source of evidence for market authorization decisions by regulatory bodies. However, explanatory approaches provide limited knowledge about how newly marketed drugs work once applied under “real-world” conditions and/or when compared with existing treatments for the same condition in clinical practice [3, 4].

The lack of generalizability in drug RCTs has led to a knowledge gap between what we know about the isolated biological effects of a pharmaceutical compound and what we know about its comparative effectiveness in daily medical practice [5–9]. In 1967, Daniel Schwarz and Joseph Lellouch published their landmark paper in which they differentiate between explanatory and pragmatic RCTs [10]. The authors noted that too often trialists were not properly addressing their research questions due to failure to match their trial design to the type of answers they were seeking. According to the authors, the explanatory approach should be used when the aim is to obtain information about whether a treatment works under ideal conditions. Hereto, a highly selected study population is required and extraneous effects (such as the placebo effect) ought to be ruled out. The pragmatic approach, on the other hand, has the aim of directly informing health care professionals by comparing treatments under the conditions they would be applied in practice (which includes extraneous effects) [11]. In pragmatic research, existing treatments can be tested against one another for their comparative effectiveness in real life, or new treatments are compared with (a variety of) usual care for a specific condition. Because of the type of questions they seek to answer, pragmatic trials generate so-called “real-world evidence” which has potential to overcome the current knowledge gap between drug efficacy and effectiveness.

Though no trial is completely explanatory nor completely pragmatic, trial designs can be assessed as being either more explanatory (idealized circumstances) or more pragmatic (resembling usual or real-world care) within a continuum [12]. A trial that does not apply strict exclusion criteria (to better reflect the real-world population), that recruits patients with no more effort than would be used to engage patients in usual care and that allows physicians considerable flexibility in how they deliver the intervention, can be called more pragmatic than explanatory.

Recent collaborative initiatives to facilitate the conduct of pragmatic trials consist of the National Institutes of Health (NIH) Health Care Systems Research Collaboratory [13] and the US Patient-Centered Clinical Research Network (PCORnet) [14]. In general terms, their mission is to increase the quality and reduce the costs of clinical research through stakeholder engagement and use of large amounts of health data. Pragmatic trials thus far have almost always been discussed as post-market authorization research. This renders pragmatic trials for many almost synonymous to pragmatic comparative effectiveness trials. However, real-world evidence on the comparative effectiveness of drugs can in principle also be collected by pragmatic trials in earlier phases of the drug life cycle. The Innovative Medicines Initiative's (IMI) multi-stakeholder GetReal Consortium has the objective of exploring new methods to incorporate real-world evidence earlier into the process of drug development to better inform health care decision-makers about the real-world effectiveness of new drugs at market authorization [15].

Though regulatory bodies do not demand real-world studies for all approved products per se, the European Medicines Agency (EMA) does support the goals of pragmatic trials through the development of so-called “adaptive pathways.” Adaptive pathways, according to the EMA, is a scientific concept for drug development which allows for early and progressive patient access to new drugs through conditional licensing, which requires real-world evidence collection to support clinical trial data through an iterative process [16]. For conceptual clarity, we refer to pragmatic trials as RCTs that are “(d)esigned for the primary purpose of informing decision-makers regarding the comparative balance of benefits, burdens and risks of a biomedical or behavioral health intervention at the individual or population level” (a definition that does not distinguish between pre- and post-market authorization research) [17]. We use real-world comparative effectiveness as a measure that can be evaluated both before and after market authorization of the tested drug, though we acknowledge that comparative effectiveness research (CER) is typically conducted with standard of care treatments.

Considering recent initiatives to implement pragmatic trials in routine health care settings—especially in earlier phases of the drug life cycle—parallel ethical evaluation appears warranted [18]. Such evaluation seems to become even more compelling as recent debate has particularly focused on the ethical acceptability of pragmatic trials in terms of altering informed consent requirements [19], the inclusion of vulnerable populations [20], determining adequate oversight practices [21], and the harms and benefits patients face in pragmatic trials [22]. In the process of articulating the ethical challenges of pragmatic trials with pharmaceutical drugs – especially when implemented in drug development – stakeholders’ views are an important source of information.

We performed a qualitative study to obtain insight into stakeholders’ views on the ethical challenges of pragmatic trials comparing pharmaceutical treatments. To trace potential ethically relevant differences between pre- and post-market authorization pragmatic trials, a secondary aim was to capture stakeholders’ attitudes towards the implementation of pragmatic trials in drug development. This study was undertaken as part of the IMI GetReal Consortium [15].

## Methods

### Study design and setting

This descriptive qualitative study aims to identify the experiences, perceptions and attitudes from the point of view of key stakeholders to explore the ethical challenges of pragmatic clinical trials investigating pharmaceutical drugs. Interviewees were identified from global stakeholders involved in the conduct of pragmatic trials and real-world studies, including stakeholders within academia, nonprofit research institutions, contract research organizations (CROs), the pharmaceutical industry, regulatory authorities, health care insurers and Health Technology Assessment (HTA) agencies as well as patient organizations. Table 1 provides a description of stakeholder characteristics. Since experience with pragmatic trials in this field is relatively scarce, respondents were identified by means of purposeful sampling. In total 42 stakeholders were approached by email for interviews; of these, two stakeholders declined an interview due to time constraints and six of them were nonresponders. Semistructured, in-depth interviews were conducted face-to-face or, when distance was a problem (e.g., for respondents located outside Europe), by telephone or through an online connection. Group interviews were set up with respondents who were involved in the same projects within one company or institution.

**Table 1:** Background of interviewed stakeholders

Respondent characteristics	Number
	34
Sex	
Male	21
Female	13
IMI GetReal member	
Yes	6
No	28
Country of professional residence	
The Netherlands	9
United Kingdom	8
Germany	5
France	2
Belgium	1
Denmark	1
United States	7
Israel	1
Experience with pragmatic trials	
Yes	23
No	11
Stakeholder group	
Pharmaceutical industry	16
Academia/nonprofit research institutions	6
Contract research organizations (CROs)	4
Health Technology Assessment (HTA)	2
Regulatory bodies	2
Patient representatives	2
Clinicians	1
Health insurers	1

### Selection of participants

An invitation email with an information sheet was sent to target participants identified through the network of the IMI GetReal Consortium, and by following recommendations from the interviewees (so-called snowball sampling) [23]. Stakeholders were first asked to describe their experience with either designing, conducting or assessing real-world studies in general or, more specifically, with pragmatic clinical trials. Subsequently, they were asked to elaborate on any relevant challenges or hurdles that were faced during the process. These challenges could either pertain to specific pragmatic design aspects as well as to more general complexities throughout the whole process of designing, conducting or assessing a pragmatic trial. When a respondent put forward a study that he or she was involved in which was relevant in terms of ethical challenges, this study was pinpointed for further enquiry if needed. Respondents were, in addition, asked to specifically reflect on implementation of pragmatic trials before regulatory approval of the test intervention. The design of the Salford Lung Study was described to stakeholders as an example of a pre-market authorization pragmatic trial (see Additional file 1) [24]. Recruitment was terminated when saturation was reached, indicating that no new thematic content was found [25].

All interviews were conducted between April and October 2014. They were conducted by a trained interviewer (SK) and took approximately 45–60 min. All interviews were audio-recorded with permission of the interviewees and transcribed verbatim. According to the Dutch Medical Research Involving Human Subjects Act, this type of study is exempt from ethical review. Verbal consent was obtained from all respondents prior to the interviews. The anonymity of respondents and institutions was maintained in the interview transcripts.

### Analysis and reporting

Transcripts of the interviews were coded in NVivo qualitative data analysis software (version 10, QSR International Pty Ltd.). Thematic analysis was used to identify ethical considerations across data sets [26]. All interview transcripts were coded by SK. For validation purposes, 14 out of 29 interviews were double-coded by two additional reviewers (GvT and AM), after which any discrepancies were discussed until consensus was reached. We used the consolidated criteria for reporting qualitative research (COREQ) checklist to guide the conduct, analysis and reporting of this study [27]. See Additional file 2 for the COREQ checklist.

## Results

Thirty-four stakeholders were interviewed in 25 individual interviews and four group interviews with a response rate of 34/42 (81 %). After analysis of the interview data, we identified four ethically relevant themes from the respondents’ views on the ethical challenges of pragmatic trials investigating pharmaceutical drugs.

### Less controlled conditions create safety concerns

Respondents believed that pragmatic trials do not have the degree of control that is required for more traditional RCTs, predominantly due to lack of a highly directive study protocol that physicians are instructed to follow. Physicians were thought to have considerable flexibility in how they prescribe the test drug in a pragmatic trial, i.e., prescribing the test drug as they would do with any newly marketed drug. It was also feared that physicians might prescribe doses beyond a drug’s label indication creating safety issues for the patients enrolled. This raised critical questions with respect to the responsibilities that investigators have towards protecting the interests of the patients enrolled in a pragmatic trial, if indeed conducted under less controlled conditions.

On pre-market authorization pragmatic trials, respondents perceived less controlled conditions to be particularly problematic, assuming that at these stages safety and efficacy data for and clinical experience with the test drug is limited. Stakeholders supposed that in a pragmatic trial minimal interference in real-world conditions is strived for, indicating that after randomization safety and efficacy follow-up is performed in accordance with usual practice. A sizeable collection of safety and efficacy data was considered a precondition before a pre-market authorization pragmatic trial could proceed ethically. Nevertheless, it was expressed that—even in the presence of sufficient efficacy and safety data—patients might not always be called in for regular check-ups and adverse events may not be recorded accurately as follow-up is left to the treating physician:

“It seems that having commitments to safety monitoring, at an individual clinical level – like, that people come back with some frequency – and also at an aggregate level through data safety monitoring, is really important… To say the obvious, you are dealing with real people who have real medical needs, and if there are alternatives available that are not what is being tested in the trial, we absolutely need to be responsible for the welfare of the people in our trial, and keep track of their disease.” (Bioethicist on pre-market authorization pragmatic trials)

### Comparison with suboptimal usual care compromises clinical equipoise

Respondents expressed that pragmatic trials could become ethically challenging when they incorporate “usual care” as a comparison group. Different interviewees referred to the SUPPORT study where the comparator arm (defined as “usual care”) consisted of a range of practices across a spectrum [28]. Respondents stated that if usual medical practice is used as a comparator arm, it may expose subjects to less than optimal medical care.

A respondent within the pharmaceutical industry highlighted discussions with regulatory authorities about including usual care treatments in a randomized trial that were not believed to constitute the “standard of care” due to either insufficient quality of the treatments or their suboptimal delivery. The respondent also expressed the view that choosing a comparator that lacks quality would make the trial results less informative:

“The aim of a pragmatic trial is to record what is happening in real life; however, you may experience that you cannot proceed ethically with a pragmatic study when routine care is delivered poorly. That is an issue we all have to think about. It is always a matter of finding a balance between the real-world and a more controlled, closely monitored setting in an explanatory sense.” (Pharmaceutical industry member with experience in clinical trial design and conduct)

### Tailored or waivers of informed consent infringes patient autonomy

Respondents stated that the real-world nature of pragmatic trials can be limited by additional requirements for research. If the informed consent procedure for a research intervention is more elaborate than the way consent is obtained for the same intervention in clinical practice, a pragmatic trial becomes less “real-world,” as was voiced by different interviewees. Patients were said to behave differently if they are aware that they are participating in a trial (also known as the Hawthorne effect). In addition, the amount of paperwork and the time needed to complete the informed consent procedure were experienced to impede recruitment.

Some clinical investigators and bioethicists believed that the informed consent procedure for a pragmatic trial for marketed products could perhaps be tailored, though this would greatly depend on the particulars of the study:

“I think that there is no question that for an unapproved product you would always need informed consent, I don’t want by implication to say that for approved products that you never do. But I think that discussions can be on the table and can be looked at by a case-to-case basis, for a pragmatic trial for an approved product.” (Bioethicist on the difference between pre- and post-market authorization pragmatic trials)

Respondents imagined that for some post-market authorization randomized research, informed consent perhaps could even be waived when the context would render it ethically acceptable. Justifications for waivers were named to be selection bias and limited generalizability of trial results. One bioethicist stated that, under certain specified circumstances, informed consent could be integrated into a routine clinic visit. A patient representative believed that when a patient’s treating physician asks for informed consent for trial participation this would potentially endanger the patient’s trust in receiving the best possible care from his or her physician. Another bioethicist questioned what waivers would exactly look like, either a complete waiver for informed consent or waivers for certain elements of the consent procedure.

Some interviewees expressed concerns that completely waiving informed consent would infringe patient autonomy as well as have a negative impact on patients’ trust in biomedical research. Some bioethicists stated that even if randomization would not meaningfully affect patients’ clinical outcome, they may have a legitimate base for preferring one study arm over the other due to expected side effects or dosing scheme. Bioethicists occasionally felt that researchers proposed waivers of consent merely out of convenience:

“Whether waivers are justified in drug trials really depends on the risks people bear, whether standard of care is withheld, whether it really is reasonably impossible to obtain consent in large patient numbers as that is what is often argued… I mean, is that truly so impossible? And is it impossible because of the large patient numbers or because patients would be unnecessarily burdened by the consent procedure? There is a difference there… And I believe that we should be really critical in reviewing waivers to assess whether it is really impossible or whether the waiver just acts as an excuse for the sake of convenience.” (Bioethicist on waivers of consent for post-market authorization pragmatic trials)

All interviewees expressed concerns for reducing informed consent requirements for unapproved products due to lack of real-world experience and the presumed limited knowledge base in terms of safety and efficacy.

### Minimal interference with real-world practice drives arms to equivalence

According to some respondents, a pragmatic trial allows a considerable degree of physician flexibility with regards to altering patients’ treatment while retaining them in the trial. Such flexibility was perceived to serve two ends: first, to ensure that patients enrolled in a pragmatic trial are treated optimally, and second, that a pragmatic trial remains as pragmatic as possible. This means that during a trial a patient can switch to an alternative treatment to the one they were initially randomized to, as would also be the case in real life. However, a clinical investigator had observed in a number of cases that allowing patients to switch in the course of their treatment inherently had driven the study arms to equivalence. In these cases, the intervention arm would then not separate from the comparator:

“I would like to emphasize again this problem of very pragmatic trials, which is the lack of separation (between arms). Among all the (published) pragmatic clinical trials (in the literature) – there aren’t that many of them compared to the number of RCTs done – but of the ones that are out there, I think only a couple out of maybe a dozen were able to differentiate the arms. Again, if you have confident physicians, intent-to-treat analytic approaches and a very pragmatic protocol, give it enough time, physicians doing what they typically do, will end up driving the arms to equivalence.” (Pharmaceutical industry member on industry-sponsored pragmatic trials)

Different respondents commented on this phenomenon, saying that in designing a pragmatic trial efforts should be directed at ensuring that the trial results will be informative. Though respondents identified this issue as a predominantly operational challenge, we additionally label it an ethical one as respondents seem to imply that such trial results do not sufficiently contribute to science and society.

## Discussion

In this qualitative study we interviewed 34 stakeholders to identify the experienced and perceived ethical challenges related to (early) implementation of pragmatic clinical trials with pharmaceutical drugs. Design choices approximating real-world conditions may be necessary to answer a pragmatic research question, yet have been shown to give rise to perceived ethical challenges in four domains: (1) less controlled conditions creating safety concerns, (2) comparison with suboptimal usual care compromising clinical equipoise, (3) tailored or waivers of informed consent infringing patient autonomy, and (4) minimal interference with real-world practice reducing the knowledge value of the results. The majority of the respondents believed that real-world evidence generation was valuable and necessary; however, pre-market authorization implementation of pragmatic trials was considered to increase ethical concerns as the investigational treatments would not yet have received regulatory approval.

The first challenge relates to the less controlled, real-world conditions in which a pragmatic trial is supposed to be conducted. Safety concerns were expressed about limited pre-trial data in pre-market authorization pragmatic trials, which exposes the underlying question of how much explanatory data needs to be available to control for risk of harm in more heterogeneous populations. Other concerns related to physicians’ flexibility in delivering the test intervention in routine practice: lack of a detailed study protocol or lack of protocol adherence was questioned to adequately protect the safety of enrolled patients, more so in pragmatic trials before market authorization. These concerns raise questions about the duties and responsibilities of investigators: what should investigators do when they suspect or observe that some patients in the test arm are not receiving optimal medical care? Though safety concerns were expressed *within* a pragmatic trial, none of the interviewees mentioned patient safety in the absence of real-world evidence *outside* a trial context. The current system moves from closely monitored trials to the use of new interventions in clinical practice with typically only minimal monitoring and limited, unstructured collection of safety data. Pragmatic trials could address this question of safety in routine care before allowing widespread use. One could thus argue that pragmatic trials are an important step towards increasing safe use of new drugs among the real-world patient population and can be viewed as a strong ethical reason to perform such trials, particularly in situations where there is no efficacy or safety data comparing two comparable treatments.

The second challenge consists of determining whether it is justified to randomize patients to different treatment patterns used in practice, especially when the trial is conducted under real-world conditions (supposedly under less control than in more traditional RCTs). It was questioned whether usual care ought to be submitted to randomized investigation when it consists of a range of treatments (each displaying a different risk-benefit profile) or when the treatment standard might be delivered suboptimally in the real-world setting. Ethically, there must exist a state of clinical equipoise about the net preferred medical treatment prior to randomizing patients to different interventions [29]; however, respondents echoed existing disputes about the adequacy of, or evidence base for, the interventions proposed for a trial. The ultimate goal of clinical trials is to meaningfully contribute to the understanding of different treatment effects, to which usual care comparisons have proven challenging [30]. Kass and colleagues state that “substantial evidence now points to the frequency and severity of the clinical harms that patients experience as a consequence of the medical errors and lack of supervision that occur in clinical care” [31]. For Kass and colleagues, however, the problem of underprotection in clinical care acts as a powerful incentive to undertake improvement efforts, such as comparative effectiveness research, directed at establishing which of two or more widely used treatments for the same indication works best for which patients.

Third, respondents acknowledged that informed consent procedures for post-market authorization pragmatic trials might not necessarily be as extensive as for pre-market authorization research. The more real-life a trial is aspired to be, the less room there seems to be for obtaining trial-specific informed consent (in accordance with Good Clinical Practice Guidelines), as was verbalized by respondents. Some investigators stated that the mere act of asking consent from patients interferes with real-life conditions. In order to be able to judge to what extent research consent is truly intrusive, we remark that is important to have an accurate understanding of how consent is obtained for a specific intervention in clinical practice. In the literature, modified consent has been suggested for certain pragmatic trials [19, 31–35]. For some pragmatic trials with standard of care treatments, even waivers have been proposed [36]. These suggestions have sparked controversy which was paralleled in the interviews, displaying concerns of infringing patient autonomy.

Lastly, it was understood that if a patient or physician enrolled in a pragmatic trial prefers an alternative treatment to the one the patient was initially randomized to, the patient can be allowed to change treatments while staying in the study. Respondents experienced the switching between study arms to drive the arms to equivalence, which to some indicated that the trial results are less informative because the comparator does not separate from the test drug. However, one could argue that if no treatment effects are observed in a pragmatic trial that allows switching, this is the real-life net effect – a finding that provides science and society with valuable answers to what in reality has no added benefit. Grobbee and Hoes have observed that there is ample confusion about the nature of pragmatic trials. They state that “crossover” from one treatment arm to the other can occur in both pragmatic *and* explanatory trials, and that this may not be problematic as long as patients are analyzed by means of *intention-to-treat* approaches [11]. Moreover, *not* allowing patients to switch treatments could lead to a delay in optimal care for the individual patients enrolled as well as make patients less satisfied with the overall treatment they received. However, if many crossovers are expected sample size might need to be increased when the clinically meaningful difference is smaller than the expected difference based on perfect protocol adherence. The observed attitudes towards switching highlight the need to specify what the value is that pragmatic trial results hold for society and how their value can be optimized.

We note that this explorative, qualitative study had some limitations. The difficulty with addressing the issues of pragmatic trials *in general* is that the term “pragmatic trial” refers to a range of RCTs across a continuum, each trial displaying different design features and each testing a different type of intervention. Due to the exploratory nature of our study we did not narrow down towards respondents what we understood to be a pragmatic trial. Thus, stakeholders’ implicit assumptions about design characteristics have likely influenced the ethical challenges foreseen. This means that for some pragmatic trials a raised challenge might be an issue, whereas in others it is not: e.g., detailed protocols have been observed in some trials that are self-described as pragmatic but in others they may be completely absent. From the interviews it also became clear that different, sometimes erroneous assumptions about a pragmatic trial were held. One respondent doubted whether a pragmatic trial entailed randomization or not. In addition, experiences, perceptions, opinions and speculation are ideally separated in the analysis; however, in practice this is difficult to do. Nevertheless, we believe that our study provides some valuable insights into the ethical issues of (early) pragmatic trials and, in addition, exposes some persistent difficulties in the discourse about pragmatic trials in terms of their definition and design features.

Pragmatic clinical trials are welcomed as a valuable means to obtaining the type of high-quality scientific evidence that has the potential to directly enhance health care decision-making [5–9, 17]. However, heated discussion still continues on when and how to do it, both practically and ethically. In a previous review of the literature, we found that different attitudes towards the moral relevance of the intertwinement of research and clinical care led to discussions about whether current clinical trial regulations are sufficient to protect the rights and interests of patients enrolled in pragmatic trials [21]. We believe that the experiences and perceptions identified in this qualitative study provide an important base for improving our understanding of the ethical complexities of pragmatic trials and their potential implementation in the drug development process. Further work in terms of methodological analysis and ethical evaluation is needed to flesh out which concerns pose truly meaningful ethical challenges and which do not.

## Conclusions

Recent collaborative initiatives are exploring ways to facilitate the (early) implementation of pragmatic trials in routine health care settings. To do so effectively and responsibly, the ethical challenges of pragmatic trials need to be identified and addressed. We performed a qualitative study among stakeholders in the field of drug research as a means to capture views of these challenges. With respect to pragmatic trials with pharmaceutical drugs, respondents perceived potential ethical challenges relating to the presumed lack of control, the use of routine care as a comparator, the need for modified informed consent and the power of a pragmatic trial to detect differences when crossover is allowed. We identified the related ethical challenges of risk assessment, evaluating the acceptability of usual care as a comparator and the tailoring of informed consent procedures as well as ensuring that the trial results have knowledge value. Further exploration of these perceived concerns and challenges is key to grasping the ethically relevant features of the whole range of pragmatic trials, from their implementation in drug development to their use in post-market authorization research.

## Additional files

Additional file 1: Characteristics of the Salford Lung Study (GSK, UK) [24]. (DOC 25 kb) Additional file 2: Consolidated criteria for reporting qualitative research (COREQ) checklist. (DOCX 23 kb)

## Abbreviations

CER: Comparative effectiveness research
COREQ: Consolidated criteria for reporting qualitative research
CRO: Contract research organization
HTA: Health Technology Assessment
IMI: Innovative Medicines Initiative
NIH: National Institutes of Health
PCORnet: Patient-Centered Clinical Research Network
RCT: Randomized controlled trial

## References

[1] Rothwell PM (2005). External validity of randomised controlled trials: “to whom do the results of this trial apply?”. Lancet.

[2] Califf RM, Zarin DA, Kramer JM, Sherman RE, Aberle LH, Tasneem A (2012). Characteristics of clinical trials registered in ClinicalTrials.gov, 2007–2010. JAMA.

[3] Treweek S, Zwarenstein M (2009). Making trials matter: pragmatic and explanatory trials and the problem of applicability. Trials..

[4] Loudon K, Zwarenstein M, Sullivan F, Donnan P, Treweek S (2013). Making clinical trials more relevant: improving and validating the PRECIS tool for matching trial design decisions to trial purpose. Trials..

[5] Eapen ZJ, Lauer MS, Temple RJ (2014). The imperative of overcoming barriers to the conduct of large, simple trials. JAMA.

[6] Tunis SR, Stryer DB, Clancy CM (2003). Practical clinical trials: increasing the value of clinical research for decision making in clinical and health policy. JAMA.

[7] Van Staa TP, Goldacre B, Gulliford M, Cassell J, Pirmohamed M, Taweel A, Delaney B, Smeeth L (2012). Pragmatic randomised trials using routine electronic health records: putting them to the test. BMJ..

[8] Chalkidou K, Tunis S, Whicher D, Fowler R, Zwarenstein M (2012). The role for pragmatic randomized controlled trials (pRCTs) in comparative effectiveness research. Clin Trials.

[9] Platt R, Kass NE, McGraw D (2014). Ethics, regulation, and comparative effectiveness research: time for a change. JAMA.

[10] Schwartz D, Lellouch J (2009). Explanatory and pragmatic attitudes in therapeutical trials. J Clin Epidemiol.

[11] Grobbee DE, Hoes AW (2015). Clinical Epidemiology: Principles, Methods and Applications for Clinical Research.

[12] Thorpe KE, Zwarenstein M, Oxman AD, Treweek S, Furberg CD, Altman DG, Tunis S, Bergel E, Harvey I, Magid DJ, Chalkidou K (2009). A pragmatic-explanatory continuum indicator summary (PRECIS): a tool to help trial designers. J Clin Epidemiol.

[13] National Institutes of Health (NIH) Collaboratory. Health Care Systems Research Collaboratory. https://www.nihcollaboratory.org/Pages/default.aspx/. Accessed 18 Aug 2016.

[14] Ali J, Califf R, Sugarman J (2016). Anticipated ethics and regulatory challenges in PCORnet: The National Patient-Centered Clinical Research Network. Account Res.

[15] IMI GetReal. New methods for RWE collection and synthesis. https://www.imi-getreal.eu/. Accessed 18 Aug 2016.

[16] European Medicines Agency (EMA). Adaptive pathways. http://www.ema.europa.eu/ema/index.jsp?curl=pages/regulation/general/general_content_000601.jsp. Accessed 18 Aug 2016.

[17] Califf RM, Sugarman J (2015). Exploring the ethical and regulatory issues in pragmatic clinical trials. Clin Trials.

[18] Sugarman J, Califf RM (2014). Ethics and regulatory complexities for pragmatic clinical trials. JAMA.

[19] McKinney RE, Beskow LM, Ford DE, Lantos JD, McCall J, Patrick-Lake B, Pletcher MJ, Rath B, Schmidt H, Weinfurt K (2015). Use of altered informed consent in pragmatic clinical research. Clin Trials.

[20] Welch MJ, Lally R, Miller JE, Pittman S, Brodsky L, Caplan AL, Uhlenbrauck G, Louzao DM, Fischer JH, Wilfond B (2015). The ethics and regulatory landscape of including vulnerable populations in pragmatic clinical trials. Clin Trials.

[21] Kalkman S, van Thiel GJMW, Grobbee DE, van Delden JJM (2015). Pragmatic randomized trials in drug development pose new ethical questions. Drug Discov Today.

[22] Ali J, Andrews JE, Somkin CP, Rabinovich CE (2015). Harms, benefits, and the nature of interventions in pragmatic clinical trials. Clin Trials.

[23] Creswell JW. Qualitative Inquiry and Research Design: Choosing among Five Traditions. 3rd ed. Thousand Oaks: SAGE Publications Ltd.; 2013.

[24] New JP, Bakerly ND, Leather D, Woodcock A (2014). Obtaining real-world evidence: the Salford Lung Study. Thorax.

[25] Guest G, Brunce A, Johnson L (2006). How many interviews are enough? An experiment with data saturation and variability. Field Methods..

[26] Boeije H. Analysis in Qualitative Research. Thousand Oaks: SAGE Publications Inc.; 2012.

[27] Tong A, Sainsbury P, Craig J (2007). Consolidated criteria for reporting qualitative research (COREQ): a 32-item checklist for interviews and focus groups. Int J Qual Health Care.

[28] Carlo WA, Finer NN, Walsh MC, Rich W, Gantz MG, Laptook AR, Yoder BA, Faix RG, Das A, Poole WK, Schibler K, Newman NS, Ambalavanan N, Frantz ID, Piazza AJ, Sánchez PJ, Morris BH, Laroia N, Phelps DL, Poindexter BB, Cotten CM, Van Meurs KP, Duara S, Narendran V, Sood BG, O’Shea TM, Bell EF, Ehrenkranz RA, Watterberg KL, Higgins RD, SUPPORT Study Group of the Eunice Kennedy Shriver NICHD Neonatal Research Network (2010). Target ranges of oxygen saturation in extremely preterm infants. N Engl J Med.

[29] Freedman B (1987). Equipoise and the ethics of clinical research. N Engl J Med..

[30] Dawson L, Zarin DA, Emanuel EJ, Friedman LM, Chaudhari B, Goodman SN (2009). Considering usual medical care in clinical trial design. PLoS Med.

[31] Kass NE, Faden RR, Goodman SN, Pronovost P, Tunis S, Beauchamp TL (2013). The research-treatment distinction: a problematic approach for determining which activities should have ethical oversight. Hastings Cent Rep.

[32] Wendler D (2015). “Targeted” consent for pragmatic clinical trials. J Gen Intern Med..

[33] Shaw D (2014). HEAT-PPCI sheds light on consent in pragmatic trials. Lancet.

[34] Kim SY, Miller FG (2014). Informed consent for pragmatic trials—the integrated consent model. N Engl J Med.

[35] Whicher D, Kass N, Faden R (2015). Stakeholders’ views of alternatives to prospective informed consent for minimal-risk pragmatic comparative effectiveness trials. J Law Med Ethics.

[36] Faden R, Kass N, Whicher D, Stewart W, Tunis S (2013). Ethics and informed consent for comparative effectiveness research with prospective electronic clinical data. Med Care..

