# Supplementary material for: Stakeholders’ views on the ethical challenges of pragmatic trials investigating pharmaceutical drugs
Source: Trials. 2016 Aug 22;17(1):419. doi: 10.1186/s13063-016-1546-3 (PMC4994208; doi:10.1186/s13063-016-1546-3)
Supplement: Additional file 1: — Characteristics of the Salford Lung Study (GSK, UK) [24]. (DOC 25 kb) [file 13063_2016_1546_MOESM1_ESM.doc]

- Open-label pre-license phase III pragmatic randomized effectiveness trial (2012-)
- Population: patients with asthma treated in general practice in Salford (United Kingdom)
- Test arm: Once-daily long-acting β2-agonist (LABA)/inhaled corticosteroid (ICS) (fluticasone furoate + vilanterol) in novel dry powder inhaler
- Comparator arm: continuation of usual asthma treatment (ICS or ICS/LABA)
- Primary outcome: improvement in asthma control (Asthma Control Test)
- Follow-up time: 12 months by means of electronic health records
- Efficacy and safety data available for more than 6400 patients from completed RCTs at the time of study initiation
