# Supplementary material for: Stakeholders’ views on the ethical challenges of pragmatic trials investigating pharmaceutical drugs
Source: Trials. 2016 Aug 22;17(1):419. doi: 10.1186/s13063-016-1546-3 (PMC4994208; doi:10.1186/s13063-016-1546-3)
Supplement: Additional file 2: — Consolidated criteria for reporting qualitative research (COREQ) checklist. (DOCX 23 kb) [file 13063_2016_1546_MOESM2_ESM.docx]

**Table S1** Consolidated criteria for reporting qualitative studies (COREQ): 32-item checklist

| **No** | **Item** | **Guide questions/description** |
| --- | --- | --- |
| **Domain 1: Research team and reflexivity** |  |  |
| Personal Characteristics |  |  |
| 1. | Interviewer/facilitator | Which author/s conducted the interview or focus group? *Shona Kalkman* |
| 2. | Credentials | What were the researcher's credentials? *MD* |
| 3. | Occupation | What was their occupation at the time of the study? *PhD student / junior researcher Medical Humanities (Ethics)* |
| 4. | Gender | Was the researcher male or female? *Female* |
| 5. | Experience and training | What experience or training did the researcher have? *In-house training provided by experienced department staff* |
| Relationship with participants |  |  |
| 6. | Relationship established | Was a relationship established prior to study commencement? *No* |
| 7. | Participant knowledge of the interviewer | What did the participants know about the researcher? *Participants received written information by e-mail about the study aims and procedure and consented by e-mail to study involvement and again at interview initiation. At the start of the interview, the goals of the research were repeated and explained in more detail, when the respondent requested this. The background and occupation of the researcher(s) was stated to respondents both by e-mail and at interview initiation.* |
| 8. | Interviewer characteristics | What characteristics were reported about the interviewer/facilitator? *Respondents were informed that the study was conducted as part of the GetReal consortium, which has the objective of exploring new methods to incorporate real world evidence earlier into the drug development process to enhance health care decision-making. Written information about the interests and aims of GetReal was sent to potential participants prior to the interview by e-mail.* |
| **Domain 2: study design** |  |  |
| Theoretical framework |  |  |
| 9. | Methodological orientation and Theory | What methodological orientation was stated to underpin the study? *Content (thematic) analysis* |
| Participant selection |  |  |
| 10. | Sampling | How were participants selected? *Purposeful sampling* |
| 11. | Method of approach | How were participants approached? *Face-to-face, by telephone and/or e-mail* |
| 12. | Sample size | How many participants were in the study? *34 respondents* |
| 13. | Non-participation | How many people refused to participate or dropped out? Reasons? *In total 42 stakeholders were approached by email for interviews; of these, two stakeholders declined an interview due to time constraints and six of them were non-responders.* |
| Setting |  |  |
| 14. | Setting of data collection | Where was the data collected? *Workplace* |
| 15. | Presence of non-participants | Was anyone else present besides the participants and researchers? *No* |
| 16. | Description of sample | What are the important characteristics of the sample? *See included table containing respondent characteristics* |
| Data collection |  |  |
| 17. | Interview guide | Were questions, prompts, guides provided by the authors? Was it pilot tested? *Stakeholders were first asked to describe their experience with either designing, executing or assessing real-world studies in general or, more specific, pragmatic trials in drug development. Subsequently, they were asked to elaborate on any relevant challenges or hurdles that were faced during the process. These challenges could either pertain to specific pragmatic design aspects as well as to more general complexities throughout the whole process of designing, executing or assessing a pragmatic trial. When a respondent put forward a study he or she was involved in which was relevant in terms of ethical challenges, this study was pinpointed for further enquiry. Stakeholders who did not have any direct experience with pragmatic trials were presented the design of the Salford Lung Study which acted as case study, upon which they were asked to further elaborate.* |
| 18. | Repeat interviews | Were repeat interviews carried out? If yes, how many? *No* |
| 19. | Audio/visual recording | Did the research use audio or visual recording to collect the data? *Yes, audio-recording* |
| 20. | Field notes | Were field notes made during and/or after the interview or focus group? *Yes, followed by verbatim transcription* |
| 21. | Duration | What was the duration of the interviews or focus group? *Approximately 45-60 minutes* |
| 22. | Data saturation | Was data saturation discussed? *Yes, among three researchers (SK, AM and GvT)* |
| 23. | Transcripts returned | Were transcripts returned to participants for comment and/or correction? *Only at request* |
| **Domain 3: analysis and findings**z |  |  |
| Data analysis |  |  |
| 24. | Number of data coders | How many data coders coded the data? *All interview transcripts were coded by SK. For validation purposes, 14 out of 29 interviews were double-coded by two additional reviewers (GvT and AM), after which any discrepancies were discussed until consensus was reached.* |
| 25. | Description of the coding tree | Did authors provide a description of the coding tree? |
| 26. | Derivation of themes | Were themes identified in advance or derived from the data? *Derived from data* |
| 27. | Software | What software, if applicable, was used to manage the data? *NVivo qualitative data analysis software (version 10, QSR International Pty Ltd.)* |
| 28. | Participant checking | Did participants provide feedback on the findings? *No* |
| Reporting |  |  |
| 29. | Quotations presented | Were participant quotations presented to illustrate the themes / findings? Was each quotation identified? *Yes* |
| 30. | Data and findings consistent | Was there consistency between the data presented and the findings? |
| 31. | Clarity of major themes | Were major themes clearly presented in the findings? *Four major themes were identified from the data: less controlled conditions creating safety concerns, comparison with usual care compromising clinical equipoise, tailored or waivers of informed consent infringing patient autonomy and minimal interference with real world practice driving study arms to equivalence.* |
| 32. | Clarity of minor themes | Is there a description of diverse cases or discussion of minor themes? *No minor themes were identified* |
